# Supplementary material for: Comparative Transcriptomics and Co-Expression Networks Reveal Tissue- and Genotype-Specific Responses of qDTYs to Reproductive-Stage Drought Stress in Rice (Oryza sativa L.)
Source: Genes (Basel). 2020 Sep 24;11(10):1124. doi: 10.3390/genes11101124 (PMC7650634; doi:10.3390/genes11101124)
Supplement: Supplementary file 1 [file genes-11-01124-s001.zip › supplementary files_DTY_20200922/Supplementary Materials_20200922.docx]

**Supplementary Materials**

**Supplementary Table legends for “Supplementary Tables_DTY_20200922.xlsx”:**

**Table S1.** Mapping results of RNA-Seq reads of Swarna and DTY-IL in flag-leaf and emerging panicle tissues under RDS and control conditions.

**Table S2.** GO enrichment analysis of the commonly down-regulated genes in DTY-IL and Swarna under RDS in the flag-leaf and emerging panicle tissues.

**Table S3**. Pathway enrichment analysis of Swarna downregulated and DTY-IL upregulated DEGs in flag-leaf under RDS.

**Table S4.** Overrepresented *cis*-acting elements of Swarna downregulated and DTY-IL upregulated DEGs in flag-leaf under RDS.

**Table S5.** GO enrichment analysis of the commonly up-regulated genes in DTY-IL and Swarna under RDS in the flag-leaf and emerging panicle tissues.

**Table S6.** Pathway enrichment analysis of Swarna downregulated and DTY-IL upregulated DEGs in emerging panicle under RDS.

**Table S7.** Overrepresented *cis*-acting elements of Swarna downregulated and DTY-IL upregulated DEGs in emerging panicle under RDS.

**Table S8.** List of different module color and sizes generated in flag-leaf and emerging panicle tissue using WGCNA.

**Table S9.** The top 10 Go terms in biological process, molecular function, and cellular component categories in FL-M1 and FL-M2 in flag-leaf, and P-M1 and P-M2 in emerging panicle tissues under RDS.

**Table S10.** The list of 140 and 102 genes with their putative functions and expression profiles in the DTY-IL and Swarna under RDS in the FL-M14 and FL-M16 modules in flag-leaf tissue.

**Table S11.** Identified hub genes in FL-M14 and FL-M16 in flag-leaf with their putative functions and expression profiles between the DTY-IL and Swarna under RDS.

**Table S12.** The list of 138 and 73 with their putative functions and expression profiles in the DTY-IL and Swarna under RDS in the P-M10 and P-M15 modules in panicle tissue.

**Table S13.** Identified hub genes in P-M10 and P-M15 in panicle with their putative functions and expression profiles between the DTY-IL and Swarna under RDS.

**Table S14.** qRT-PCR primers used in the study.

**Table S15.** Information on the introgressed chromosome segments and the differentially expressed genes in the DTY1.1-IL compared with Swarna under control and reproductive-drought stress conditions and overlapping *qDTY’s* in flag-leaf and panicle tissues.

**Table S16.** List of DEGs in the *qDTY1.1* region across the different pairwise comparisons in flag-leaf and emerging panicle transcriptomes under RDS.

**Table S17.** Distinct *cis* regulatory elements in the 2 kb upstream promoter region of LOC_Os01g67030 in Nipponbare, MH63, and N22 sequences.

**Supplementary Figure legends:**

**Figure S1**. Climate data and soil water content during water stress experiment.

**Figure S2**. Quality control assessment for 16 flag-leaf tissue samples. Visualization of *vst*-transformed values through heatmap (A) and clustering by Euclidean distance (B) was used to display similarity and dissimilarity between samples. PCA plot (C) was used for visualizing the overall effect of experimental covariates and batch effects and boxplot of Cook’s distances (D) were used to check for sample consistency. IL = DTY-IL.

**Figure S3**. Quality control assessment for 15 panicle tissue samples, with one Swarna control outlier removed (See **Figure S4**). Visualization of *vst*-transformed values through heatmap (A) and clustering by Euclidean distance (B) was used to display similarity and dissimilarity between samples. PCA plot (C) was used for visualizing the overall effect of experimental covariates and batch effects and boxplot of Cook’s distances (D) were used to check for sample consistency. IL = DTY-IL.

**Figure S4**. Quality control assessment for 16 panicle tissue samples. Visualization of *vst*-transformed values through heatmap (A) and clustering by Euclidean distance (B) was used to display similarity and dissimilarity between samples. PCA plot (C) was used for visualizing the overall effect of experimental covariates and batch effects and boxplot of Cook’s distances (D) were used to check for sample consistency. The sample dendrogram (B) and PCA (C) indicate sample Swana.Control 3 as an obvious outlier and was removed prior to DE analysis (See **Figure S3**). IL = DTY-IL.

**Figure S5**. DEGs identification. Mean-difference plots showing the result of the different pairwise differential expression analysis for flag-leaf and panicle tissues, respectively. The X-axis represents the mean of normalized counts and the Y-axis represents the fold change (on a log_2_ scale). Red dots indicate an adjusted *p*-value of less than 0.05. Red dots outside of the blue line indicate DEGs for which the log_2_ fold change was significantly higher than +/- 1. IL = DTY-IL.

**Figure S6**. Venn diagrams of differentially expressed genes (DEGs) upregulated (A) and downregulated (B) in flag-leaf as well as upregulated (C) and downregulated D) in panicle tissues at FDR adjusted *P*-value < 0.05 and -1 ≤ log_2_-ratio ≤ +1 (fold change ≥ 2. SWAC = Swarna control, SWAD = Swarna under RDS, ILC = DTY-IL control, ILD = DTY-IL under RDS.

**Figure S7**. Flag-leaf significant GO terms of biological processes that are down-regulated in Swarna (A) and up-regulated in DTY-IL (B) under RDS. The X-axis represents the significant GO terms and the Y-axis represents the number of DEGs for each significant GO terms. IL = DTY-IL.

**Figure S8**. Panicle significant GO terms of biological processes that are down-regulated in Swarna (A) and up-regulated in DTY-IL (B) under RDS. The X-axis represents the significant GO terms and the Y-axis represents the number of DEGs for each significant GO terms. IL = DTY-IL.

**Figure S9.** MapMan overview of the DEGs of interest in DTY-IL and Swarna under RDS. (A) The cell function overview in DTY-IL and (B) Swarna in the flag-leaf tissue, and (C) DTY-IL and (D) Swarna in the panicle tissue. The DEGs were binned to the MapMan functional categories. The values are the log_2_ fold changes. The upregulated and downregulated genes are represented with blue and red

**Figure S10**. Identification of gene co-expression modules in the flag-leaf (A-C) and panicle (D-F) transcriptome under RDS. Properties and topologies of gene co-expression networks in flag-leaf and panicle tissue under RDS.

(A &D) Selection of soft-thresholding power in WGCNA. The y-axis shows the scale-free topology index as a function of the Soft Threshold on the x-axis. The graph is reaching a saturation point at threshold ß value = 6 in flag-leaf (A) and ß value = 9 and panicle (D) networks.

(B & E) Hierarchical cluster dendrogram showing co-expression modules from WGCNA in flag-leaf (B) and panicle (E) tissues, respectively. The *y*-axis denotes the co-expression distance and the *x*-axis corresponds to genes. Genes were clustered based on a dissimilarity measure (1-TOM). Dynamic tree cutting was applied with a 0.15 threshold to identify modules by dividing the dendrogram at significant branch points. The branches correspond to modules of highly interconnected groups of genes. Modules corresponding to branches are displayed with different colors in the horizontal bar immediately below the dendrogram, with gray representing unassigned genes. Each vertical line a.k.a “leaf” in the tree represents a gene.

(C & F) Meta-module identification. The module network dendrogram was constructed by the hierarchical clustering of module eigengene distances in the flag-leaf and panicle networks. The horizontal line represents the threshold (0.15) used for defining the meta-modules. Branches of the dendrogram (the meta-modules) group together eigengenes that are positively correlated.

**Figure S11**. Bar graphs and Heatmaps of FL-M1 and FL-M2 in flag-leaf, and P-M1 and P-M2 in panicle tissues under RDS, which correspond to a similar expression profile of both genotypes to drought. Bar plot of the module eigengene across different samples in of FL-M1 and FL-M2 in flag-leaf (A & B) and P-M1 and P-M2 in panicle (C & D) tissues, respectively. The 4 different pairwise comparisons and the interaction term were used to assess changes in expression profiles. The X-axis represents the different samples across 4 different groups. The Y-axis corresponds to the Eigengene Value. The Eigengene Value can be considered a representative of the gene expression profiles in a module. Heatmaps showing gene expression levels of the genes and the number of genes within turquoise and blue modules in flag-leaf (A & B) and panicle (C & D) tissues, respectively across the different samples. Each column represents different samples. Each row corresponds to one gene in the module. Red is a positive expression and blue is a negative expression profile.

**Figure S12**. Major biological processes and over-represented GO Slim descriptions of drought-responsive FL-M14 (A) and FL-M16 (B) in flag-leaf tissue under RDS.

**Figure S13**. Hub genes from the drought-responsive modules in the flag-leaf. The identified hub genes in FL-M14 (A) and FL-M16 (B) between DTY-IL and Swarna under RDS. The bar graphs show the Log_2_-ratio of each hub genes between the DTY-IL and Swarna under RDS in the Y-axis and the specific hub gene on the X-axis.

**Figure S14**. Major biological processes and over-represented GO Slim descriptions of drought-responsive P-M10 (A) and P-M15 (B) in panicle tissue under RDS.

**Figure S15**. Hub genes from the drought-responsive modules in the panicles. The identified hub genes in P-M10 (A) and P-M15 (B) between DTY-IL and Swarna under RDS. The bar plots show the Log_2_-ratio of each hub genes between the DTY-IL and Swarna under RDS in the Y-axis and the specific hub gene on the X-axis.

**Figure S16.** The tissue-specific expression under RDS. Consensus network matrix for the flag-leaf and panicle networks. The X-axis represents all the 23 panicle modules with the corresponding number of genes for each module. The Y-axis represents all the 21 flag-leaf modules with the corresponding number of genes for each module. Numbers beside the module number are the total gene counts for that module. Numbers inside the matrix are the number of genes common between the flag-leaf and panicle modules. Red numbers have significant overlap in gene count based on Fisher’s exact test with the –log(p) of the p-value encoding the coloring (0 = -log(1) and 50 = -log(1E-50)). FL ME = Flag-leaf module; PAN ME = Panicle module. ME0 = grey module for both tissues that was not included as the genes in this module don’t fit anywhere else.

**Figure S17**. qRT-PCR validation of candidate genes. The selected genes from different pairwise comparison in (A) flag-leaf and (B) panicle tissues under the reproductive-stage drought stress are shown in heat-maps representing their expression profile. RNA-Seq values used are the calculated log_2_ fold change for each contrast. The scale from qPCR represents log_2_ fold change in expression. *ELF* and *ATU* were used as an endogenous control for flag-leaf and panicle tissues respectively.

**Figure S18.** Comparison of gene structure including the 2-kb upstream region of AuxRe. The 2-kb upstream and full-length sequence of AuxRe in Nipponbare was used to lift over the corresponding sequences of AuxRe in MH63v2 and N22v2 genomes via blastn. Organization of exons (blue box), introns, and UTRs (gray box) of AuxRe (LOC_Os01g67030) gene in the gene body and the 2-kb upstream sequences of the gene are shown for Nipponbare, MH63v2 (an indica reference genome used as a proxy for the closely related Swarna), and N22v2, representing *Japonica, Indica,* and *Ausboro* genomes respectively. The alignment of the promoter and the full-length genomic region was done using Blastn. Gene structure annotation was present in Nipponbare (RGAP 7) and MH63v2 (RIGW) while it was predicted in the N22v2 using FGENESH. Non-synonymous SNP’s (in color red texts) were determined relative to the Nipponbare sequence. *FGENESH prediction; **Different start codon in FGENESH prediction; ***Alignment result through Blastn in NCBI Blast. CDS = coding sequence; UTR = untranslated region; ATG = start codon; TGA = stop codon; TSS = transcription start site; TTS = transcription termination site.

**Figure S19**. Multiple peptide sequence alignment of LOC_Os01g67030 from Nipponbare, MH63v2 (an indica reference genome used as a proxy for the closely related Swarna), N22_v2, and a clone amplified from the DTY-IL parent N22 (N22_Sanger). Nipponbare sequence was obtained from the Rice Genome Annotation Project (RGAP 7), while MH63v2 sequence was obtained from the Rice Information Gateway (RIGW). Predictions in N22v2 were made in FGENESH. Protein alignment was done using Clustal Omega using default parameters with ClustalW output format. Conserved domains search was done through CD-search in NCBI with default settings. The DOMON_DOH and Cyt_b561_FRRS1_like conserved domains were underlined. Note: The Sanger sequencing result of N22 validates the SNPs and Indels in the predicted AA of N22_v2. Nonsynonymous SNPs and a Glycine deletion unique to N22v2 were highlighted (red rectangular box). *, identical residues.

**Figure S20**. Multiple peptide sequence alignment of LOC_Os03g03510 in Nipponbare, MH63, and N22 sequences. Nipponbare sequence was obtained from the Rice Genome Annotation Project (RGAP 7), while MH63v2 sequence was obtained from the Rice Information Gateway (RIGW). Predictions for N22v2 were made in FGENESH. Protein alignment was done using Clustal Omega using default parameters with ClustalW output format. Conserved domains search was done through CD-search in NCBI with default settings. The STKc_SnRK3 and CIPK_C conserved domains were underlined. The extended C-terminal region (red rectangular box) in N22v2 was highlighted. *, identical residues.

**Figure S21.** Model of suggested DTY-IL dependent drought tolerance mechanism. (A) In DTY1.1-IL in flag leaves (green), maintained, and drought-adapted cell wall homeostasis (solid green bricks) under drought (no water and sun icons) supports sustained photosynthetic activity (large green chloroplast icon). This, in turn, allows for strong source strength and the allocation of energy and carbon in the form of photo-assimilates to emerging panicles. A strong sink is established, and energy and carbon available for Redox-homeostasis  (Redox HS), protective secondary metabolism (Sec Met), and high rates of protein turnover (Protein TO) enable the emerging panicle to cope with drought stress and remain fertile, ultimately leading to successful grain filling and drought-tolerant yield (DTY). This is proposed to be in part accomplished through efficient source-sink signaling (blue arrow) suggested to be modulated through the identified candidate genes. (B) In Swarna flag leaves (green), failure to maintain cell wall integrity (wavy green bricks) under drought results in leaf rolling and impaired photosynthetic activity (small chloroplast icon). This, in turn negatively affects source strength and severely limits the allocation of energy and carbon in the form of photo-assimilates to emerging panicles. Consequently, the merging panicle is in starvation mode and fails to establish a strong sink. There is not sufficient carbon energy available to successfully counter drought stress, and the results are sterility and failure to fill grain and yield under drought. This is proposed to be in part due to failure to modulate source-sink signaling in response to drought stress (yellow lightning across blue arrow) and unfavorable alleles on the identified candidate genes suggested to contribute.

**Supplementary figures**


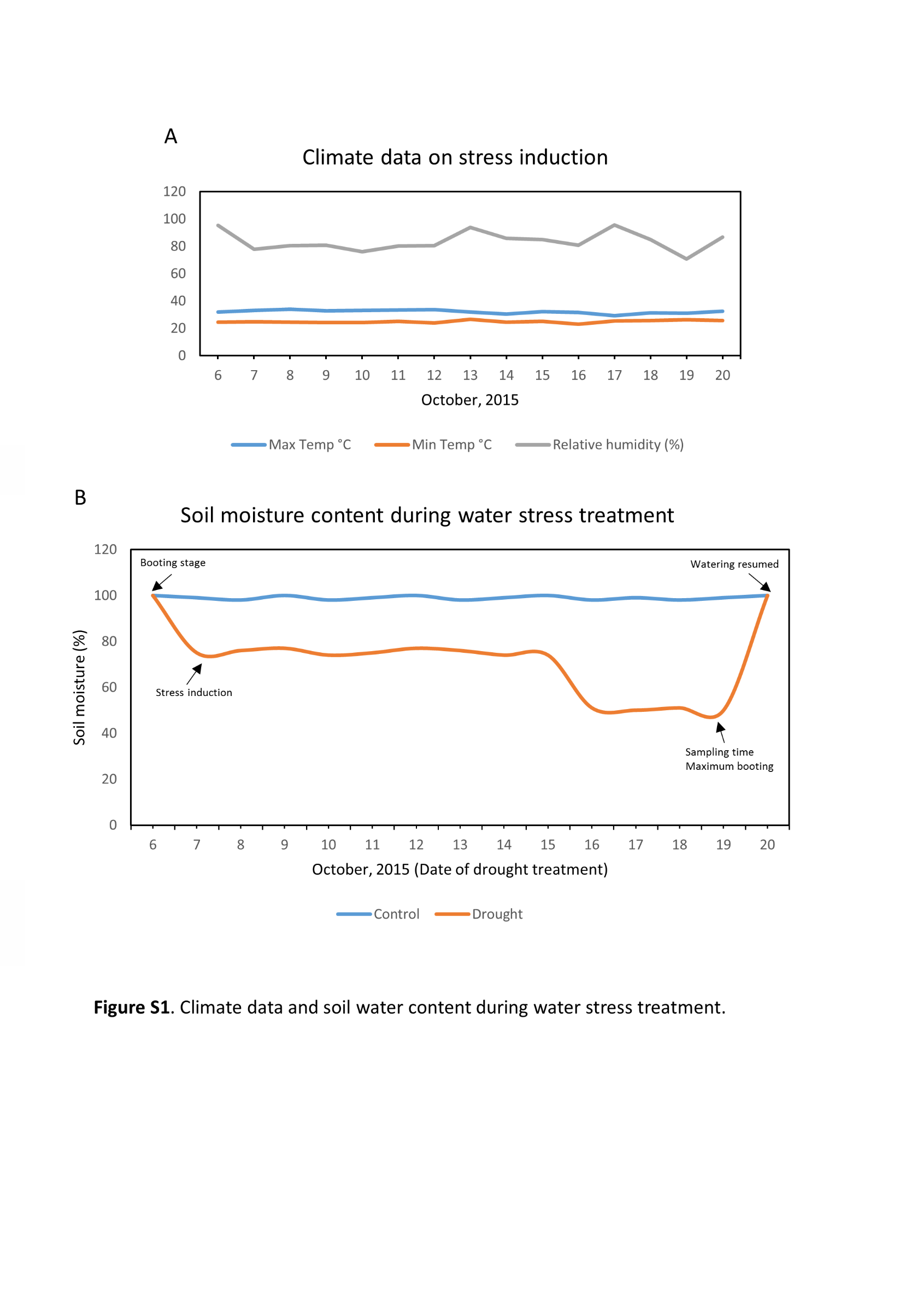


**Figure S1**. Climate data (A) and soil water content (B) during water stress experiment.


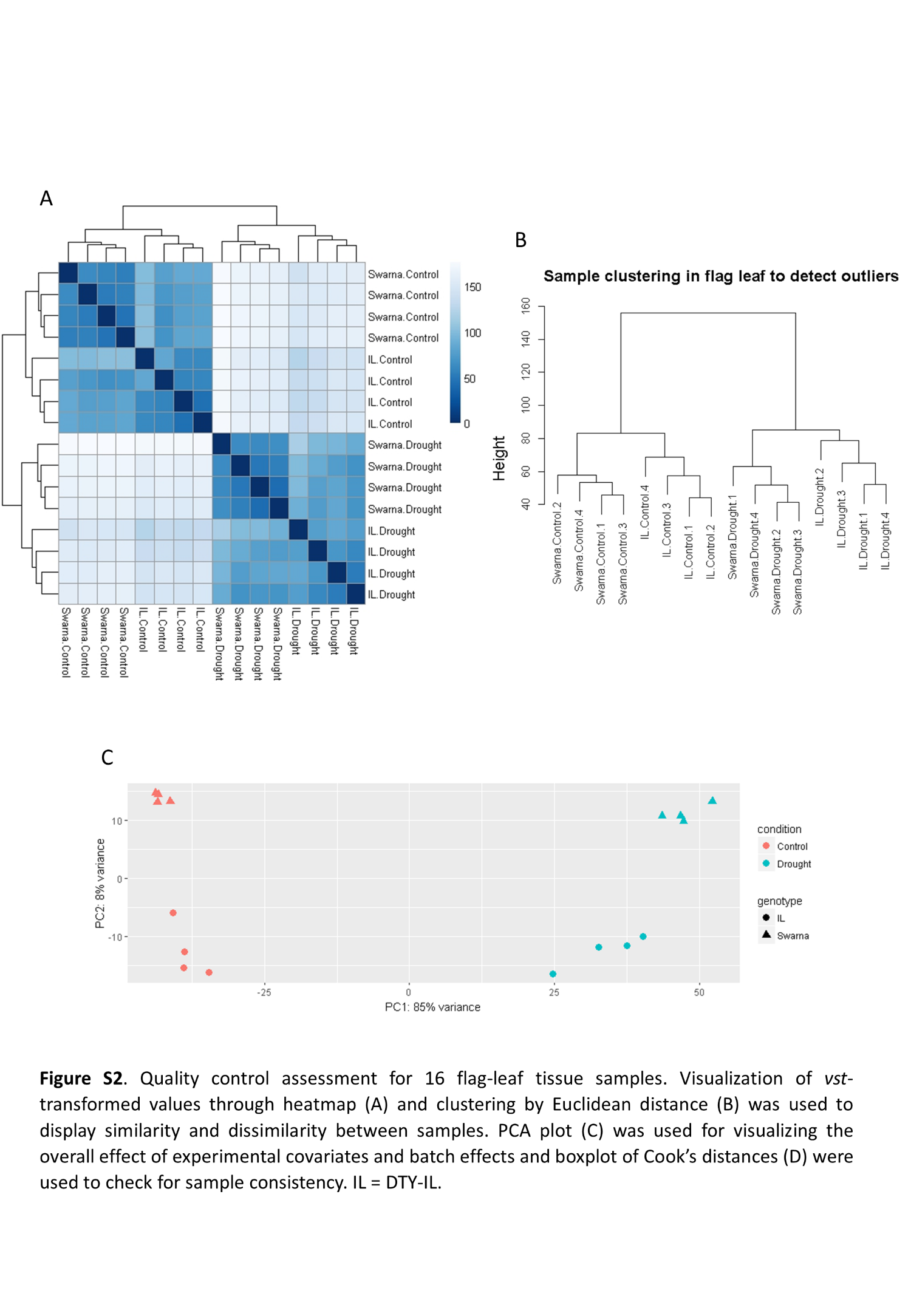


**Figure S2**. Quality control assessment for 16 flag-leaf tissue samples. Visualization of *vst*-transformed values through heatmap (A) and clustering by Euclidean distance (B) was used to display similarity and dissimilarity between samples. 2D PCA plot (C) was used for visualizing the overall effect of experimental covariates IL = DTY-IL.


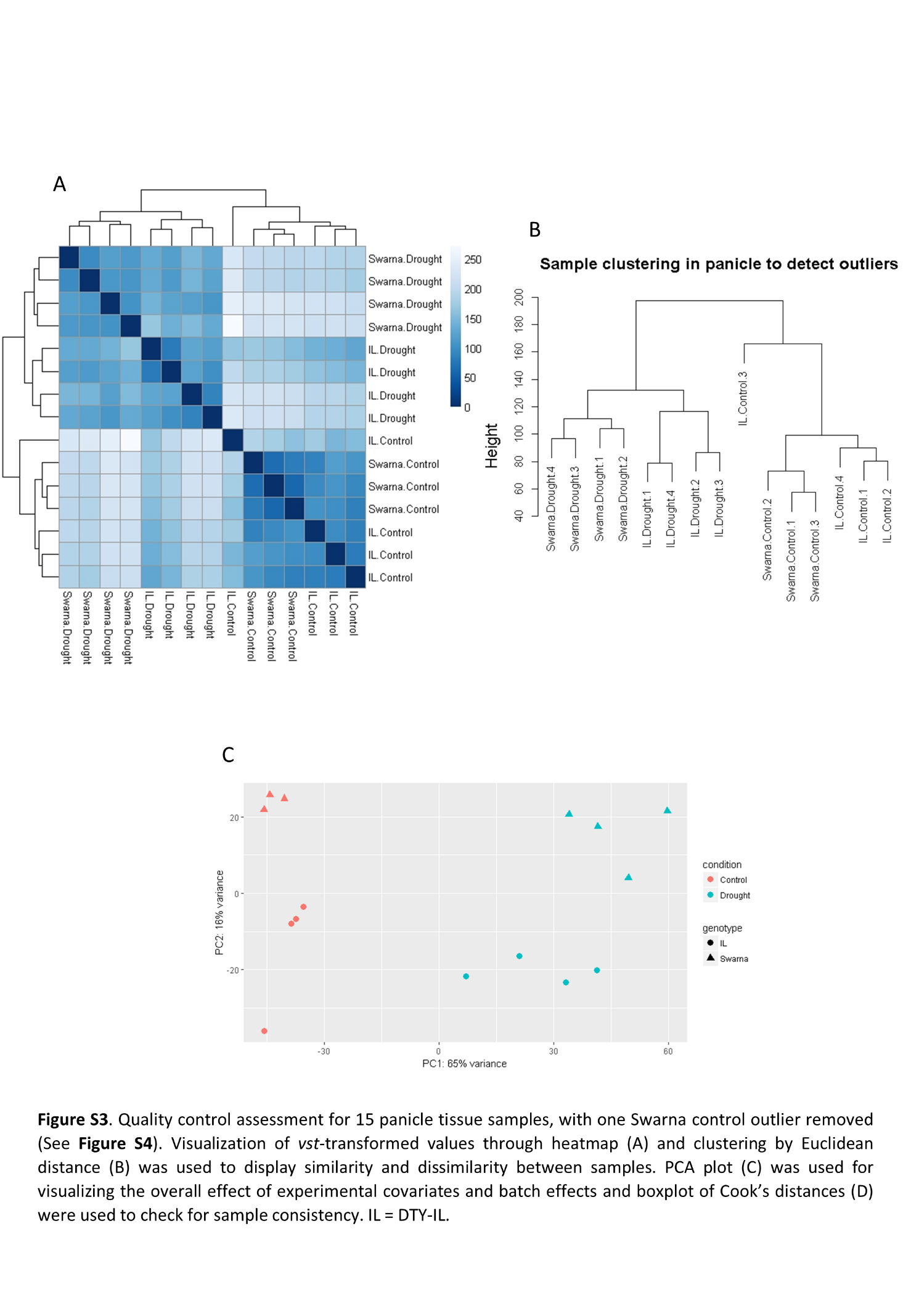


**Figure S3**. Quality control assessment for 15 panicle tissue samples. Visualization of *vst*-transformed values through heatmap (A) and clustering by Euclidean distance (B) was used to display similarity and dissimilarity between samples. 2D PCA plot (C) was used for visualizing the overall effect of experimental covariates IL = DTY-IL.


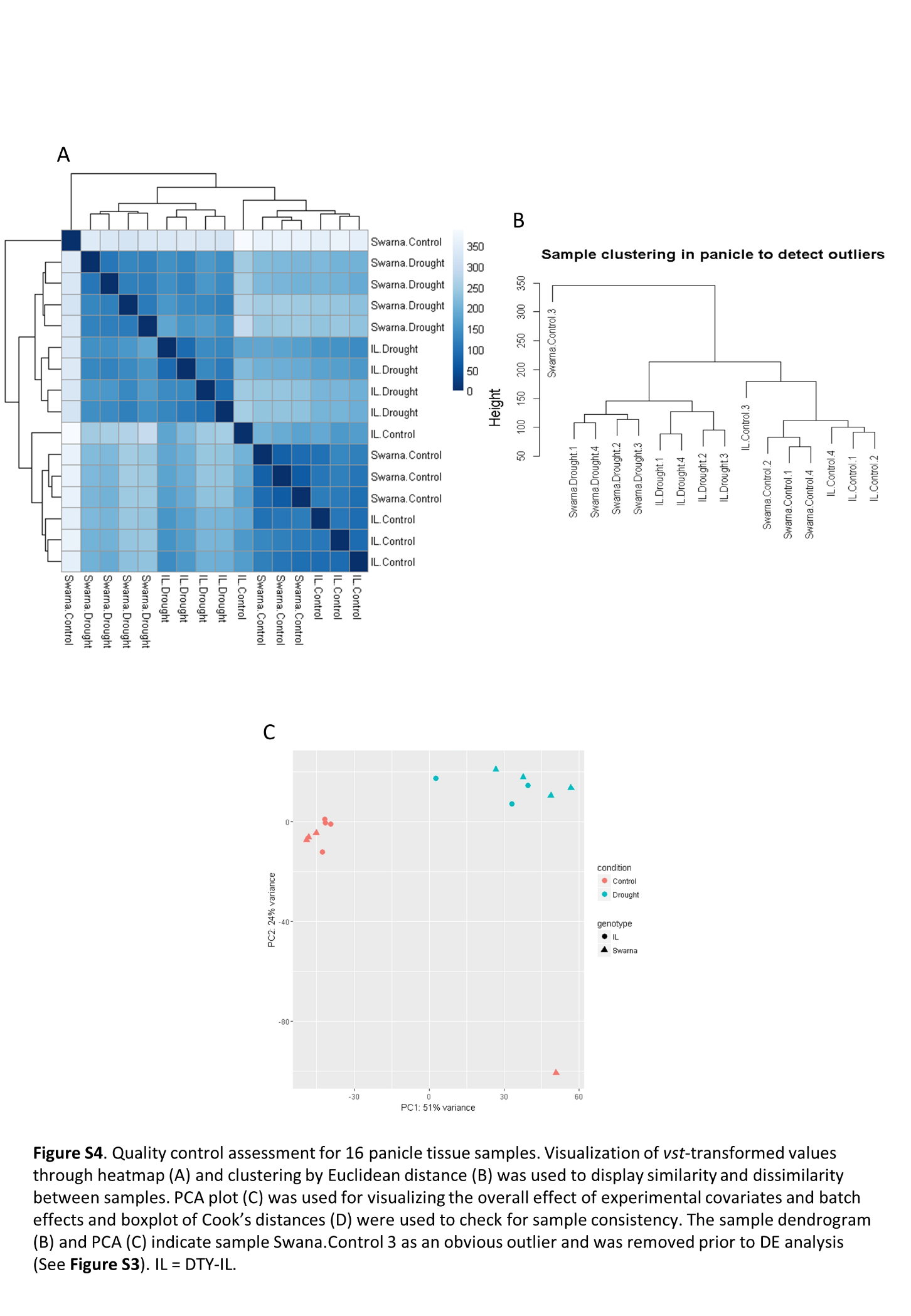


**Figure S4**. Quality control assessment for 16 panicle tissue samples. Visualization of *vst*-transformed values through heatmap (A) and clustering by Euclidean distance (B) was used to display similarity and dissimilarity between samples. PCA plot (C) was used for visualizing the overall effect of experimental covariates. The sample dendrogram (B) and PCA (C) indicate sample Swana.Control 3 as an obvious outlier and was removed prior to DE analysis (See **Figure S2 and S3**). IL = DTY-IL.

**
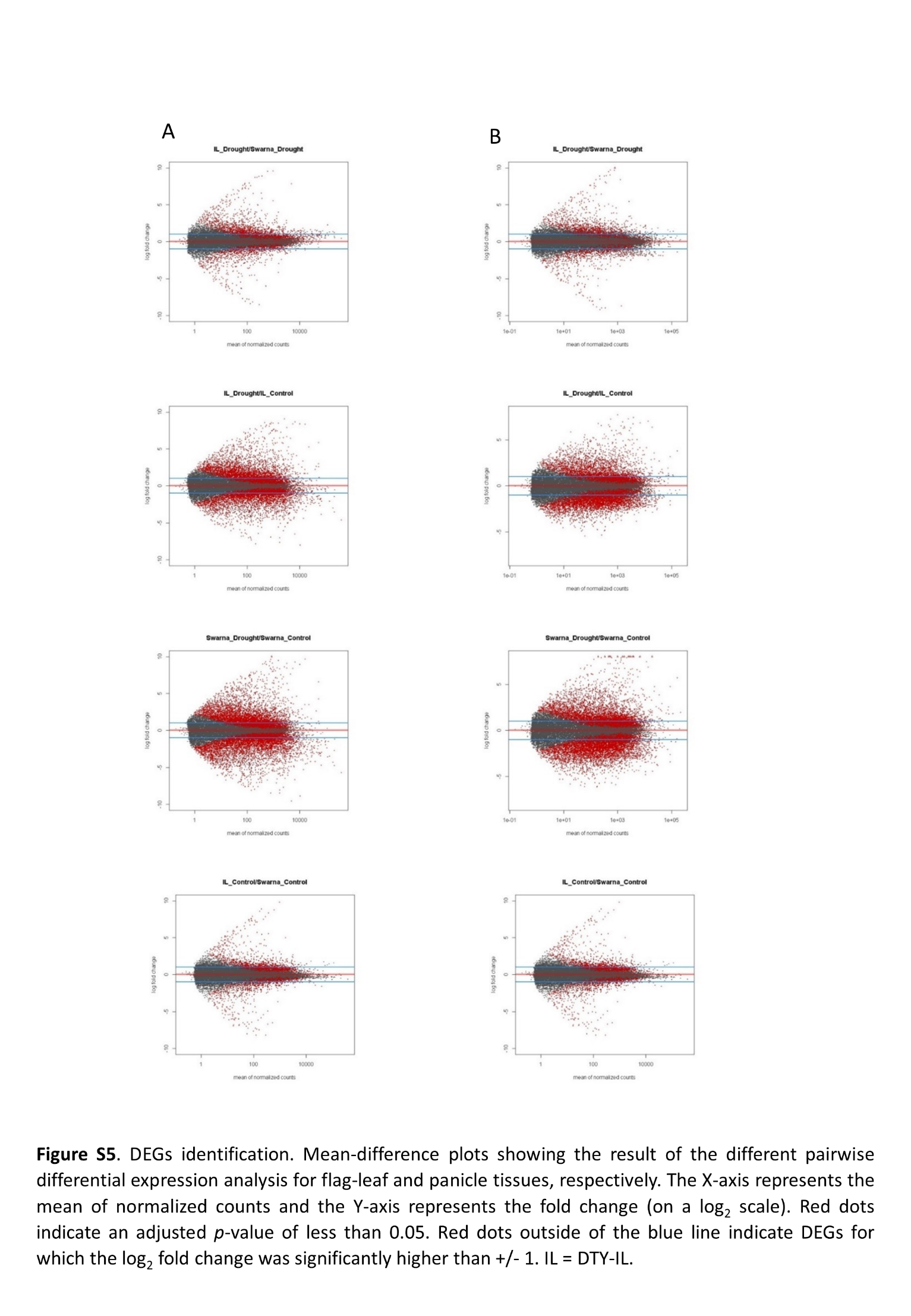
**

**Figure S5**. DEGs identification. Mean-difference plots showing the result of the different pairwise differential expression analysis for (A) flag-leaf and (B) panicle tissues, respectively. The X-axis represents the mean of normalized counts and the Y-axis represents the fold change (on a log_2_ scale). Red dots indicate an adjusted *p*-value of less than 0.05. Red dots outside of the blue line indicate DEGs for which the log_2_ fold change was significantly higher than +/- 1. IL = DTY-IL.


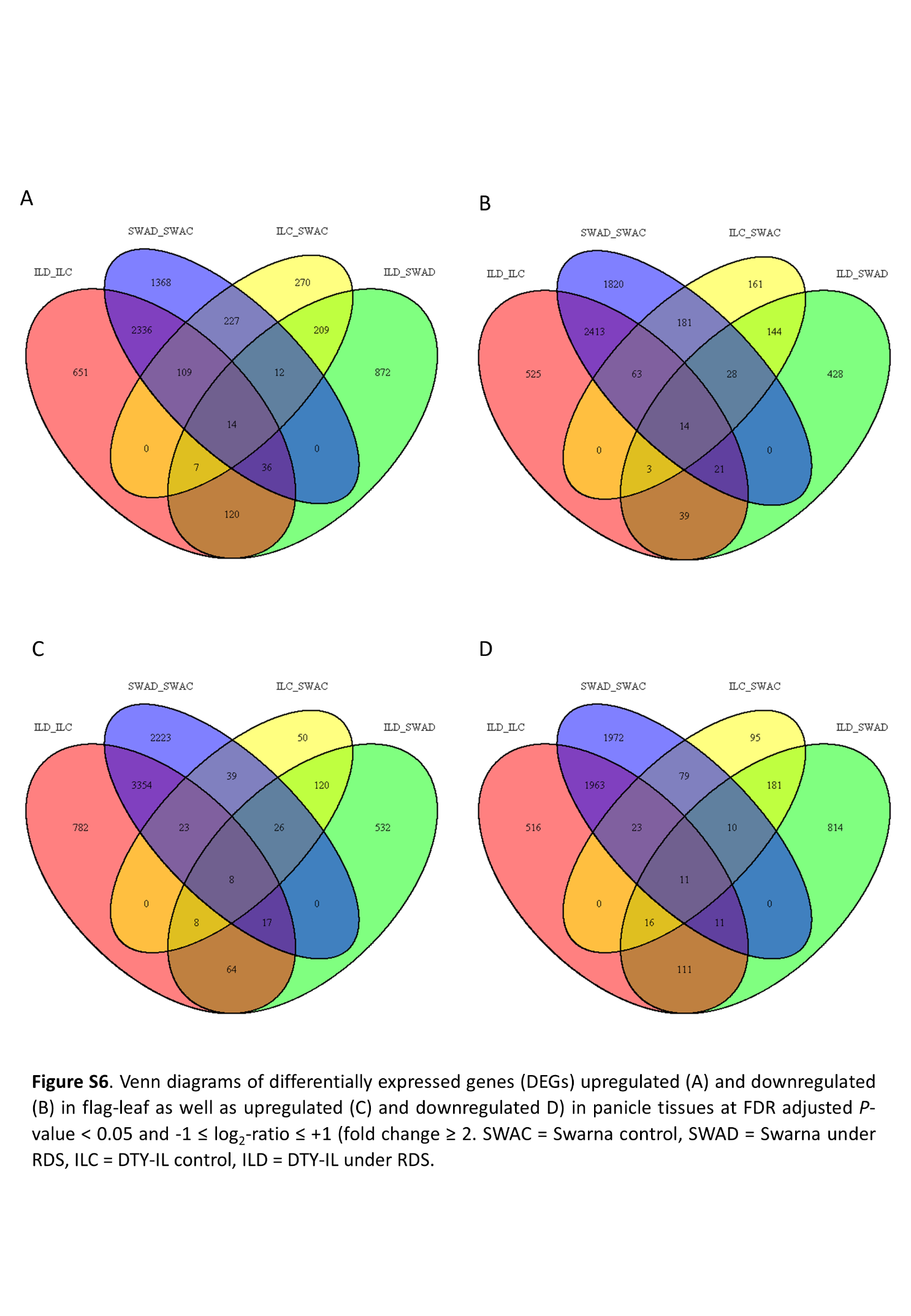


**Figure S6**. Venn diagrams of differentially expressed genes (DEGs) upregulated (A) and downregulated (B) in flag-leaf as well as upregulated (C) and downregulated D) in panicle tissues at FDR adjusted *P*-value < 0.05 and -1 ≤ log_2_-ratio ≤ +1 (fold change ≥ 2. SWAC = Swarna control, SWAD = Swarna under RDS, ILC = DTY-IL control, ILD = DTY-IL under RDS.


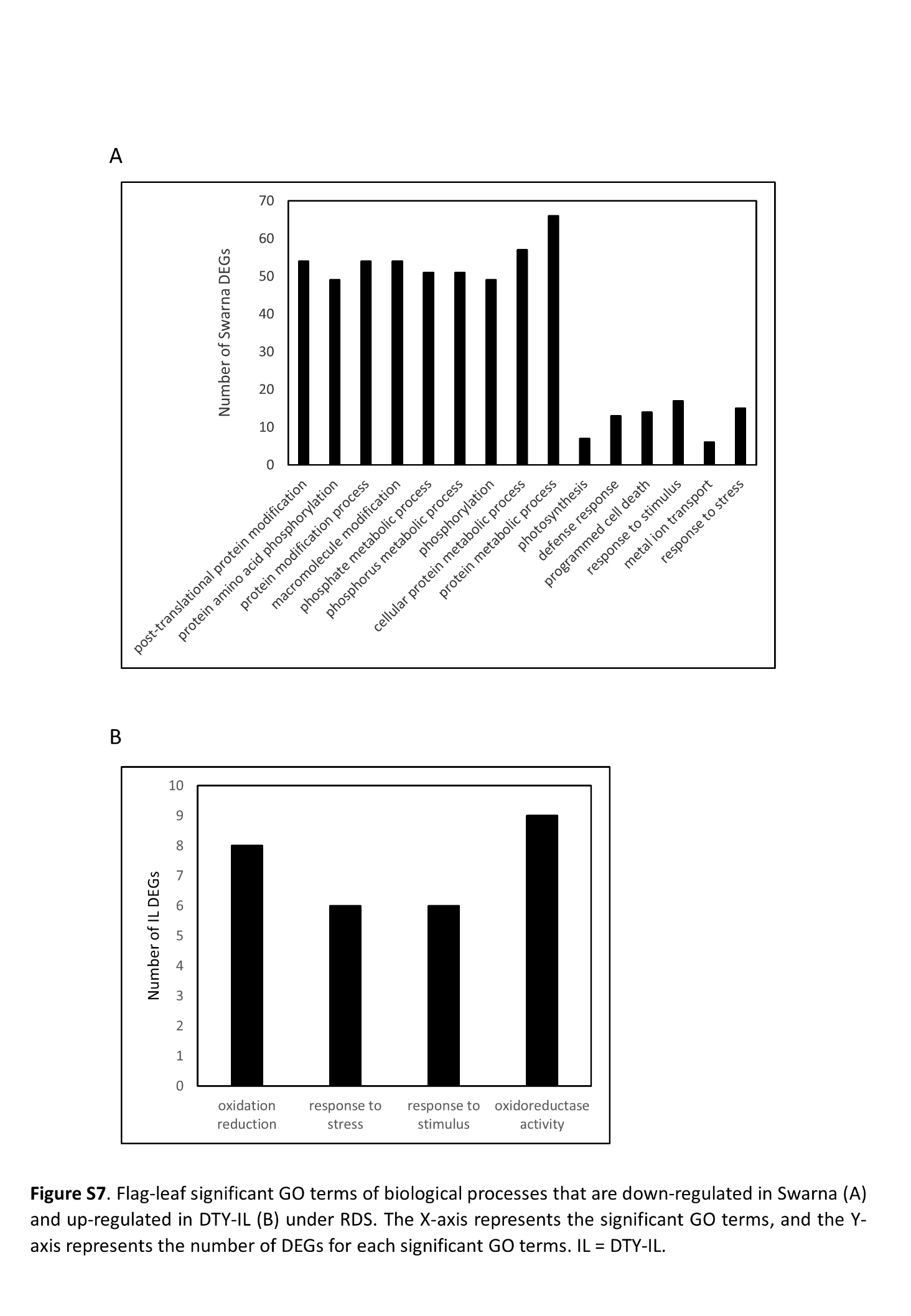


**Figure S7**. Flag-leaf significant GO terms of biological processes that are down-regulated in Swarna (A) and upregulated in IL (B) under RDS. The X-axis represents the significant GO terms and the Y-axis represents the number of DEGs for each significant GO terms. IL = DTY-IL.


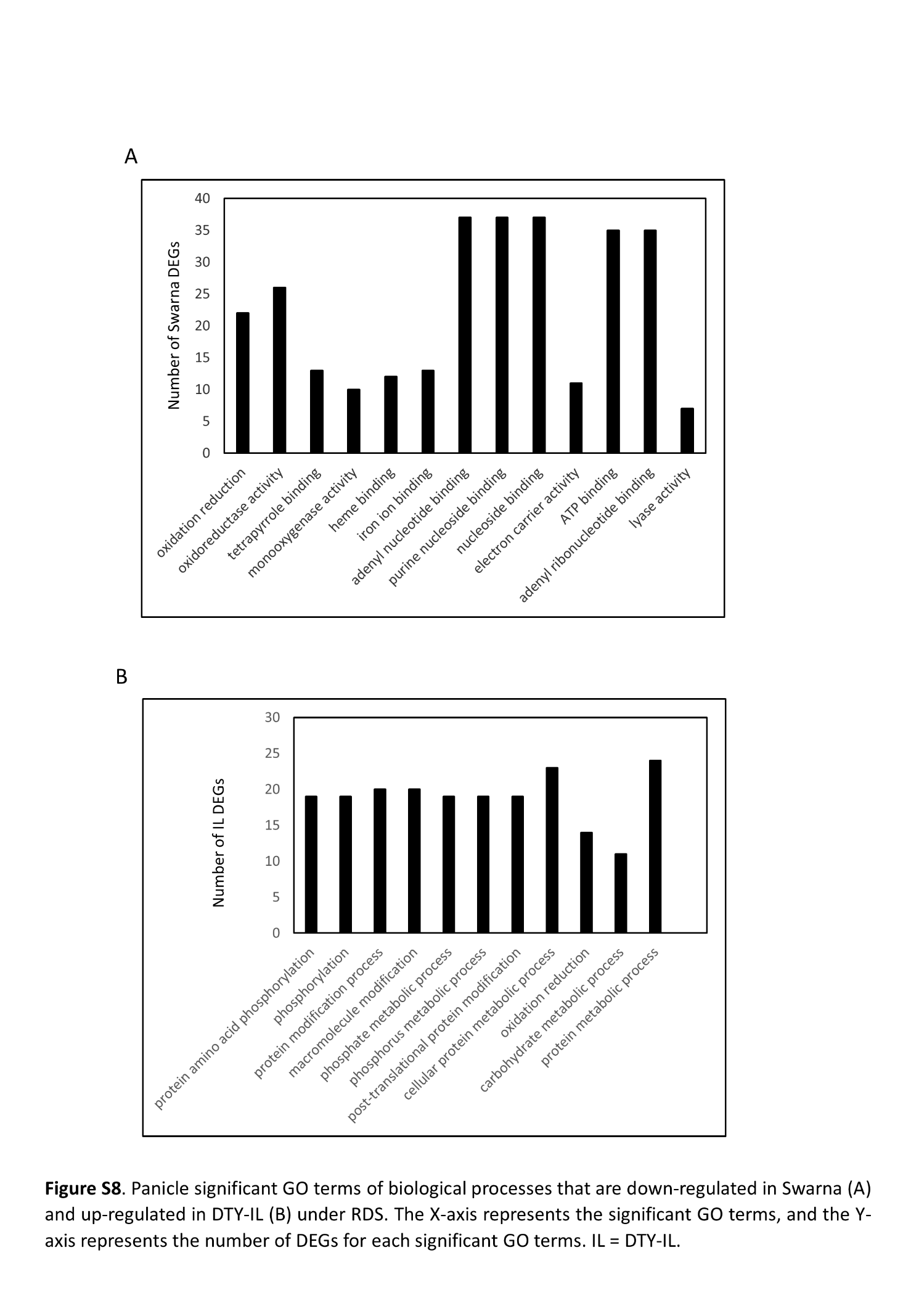


**Figure S8.** Panicle significant GO terms of biological processes that are downregulated in Swarna (A) upregulated in IL (B) under RDS. The X-axis represents the significant GO terms and the Y-axis represents the number of DEGs for each significant GO terms. IL = DTY-IL.

**
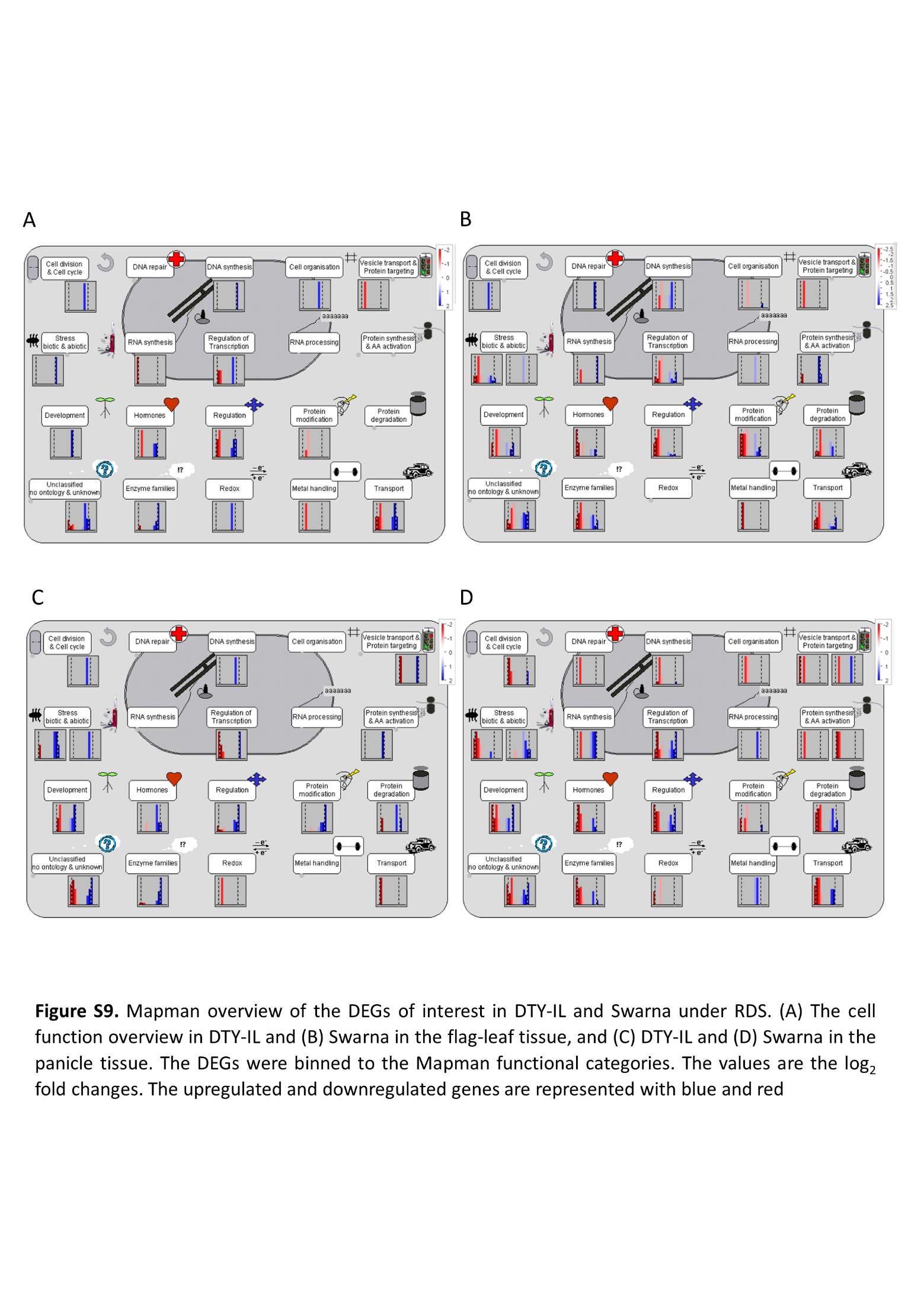
**

**Figure S9.** MapMan overview of the DEGs of interest in DTY-IL and Swarna under RDS. (A) The cell function overview in Swarna and (B) DTY-IL in the flag-leaf tissue, and (C) Swarna and (D) DTY-IL in the panicle tissue. The DEGs were binned to the MapMan functional categories. The values are the log_2_ fold changes. The upregulated and downregulated genes are represented with blue and red color respectively.


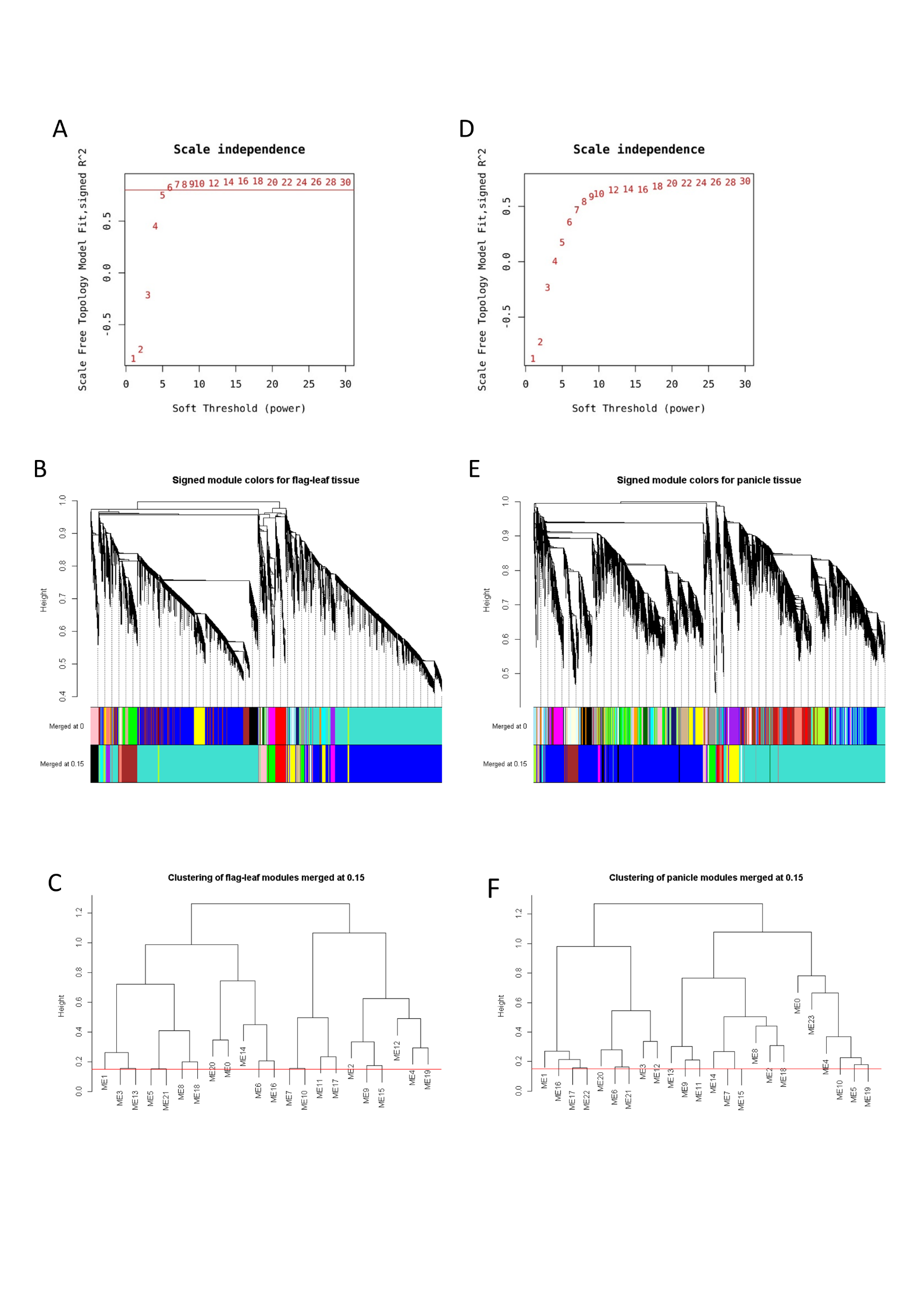


**Figure S10**. Identification of gene co-expression modules in the flag-leaf (A-C) and panicle (D-F) transcriptome under RDS. Properties and topologies of gene co-expression networks in flag-leaf and panicle tissue under RDS. (A &D) Selection of soft-thresholding power in WGCNA. The y-axis shows the scale-free topology index as a function of the Soft Threshold on the x-axis. The graph is reaching a saturation point at threshold ß value = 6 in flag-leaf (A) and ß value = 9 and panicle (D) networks. (B & E) Hierarchical cluster dendrogram showing co-expression modules from WGCNA in flag-leaf (B) and panicle (E) tissues, respectively. The *y*-axis denotes the co-expression distance and the *x*-axis corresponds to genes. Genes were clustered based on a dissimilarity measure (1-TOM). Dynamic tree cutting was applied with a 0.15 threshold to identify modules by dividing the dendrogram at significant branch points. The branches correspond to modules of highly interconnected groups of genes. Modules corresponding to branches are displayed with different colors in the horizontal bar immediately below the dendrogram, with gray representing unassigned genes. Each vertical line a.k.a “leaf” in the tree represents a gene. (C & F) Meta-module identification. The module network dendrogram was constructed by the hierarchical clustering of module eigengene distances in the flag-leaf and panicle networks. The horizontal line represents the threshold (0.15) used for defining the meta-modules. Branches of the dendrogram (the meta-modules) group together eigengenes that are positively correlated.


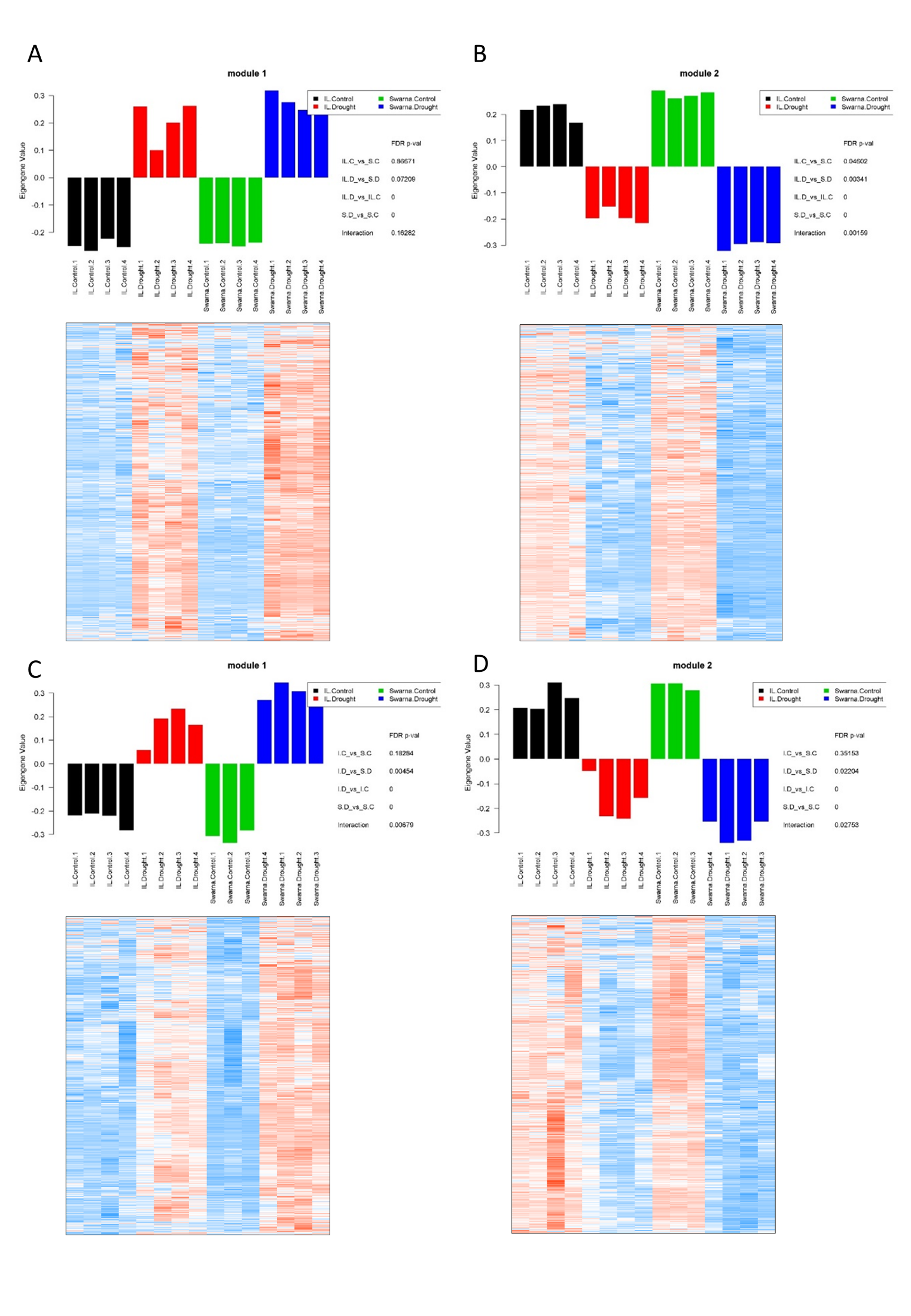


**Figure S11**. Bar graphs and Heatmaps of FL-M1 and FL-M2 in flag-leaf, and P-M1 and P-M2 in panicle tissues under RDS, which correspond to a similar expression profile of both genotypes to drought. Bar plot of the module eigengene across different samples in of FL-M1 and FL-M2 in flag-leaf (A & B), and P-M1 and P-M2 in panicle (C & D) tissues, respectively. The 4 different pairwise comparisons and the interaction term were used to assess changes in expression profiles. The X-axis represents the different samples across 4 different groups. The Y-axis corresponds to the Eigengene Value. The Eigengene Value can be considered a representative of the gene expression profiles in a module. Heatmaps showing gene expression levels of the genes and the number of genes within turquoise and blue modules in flag-leaf (A & B) and panicle (C & D) tissues, respectively across the different samples. Each column represents different samples. Each row corresponds to one gene in the module. Red is a positive expression and blue is a negative expression profile.

**
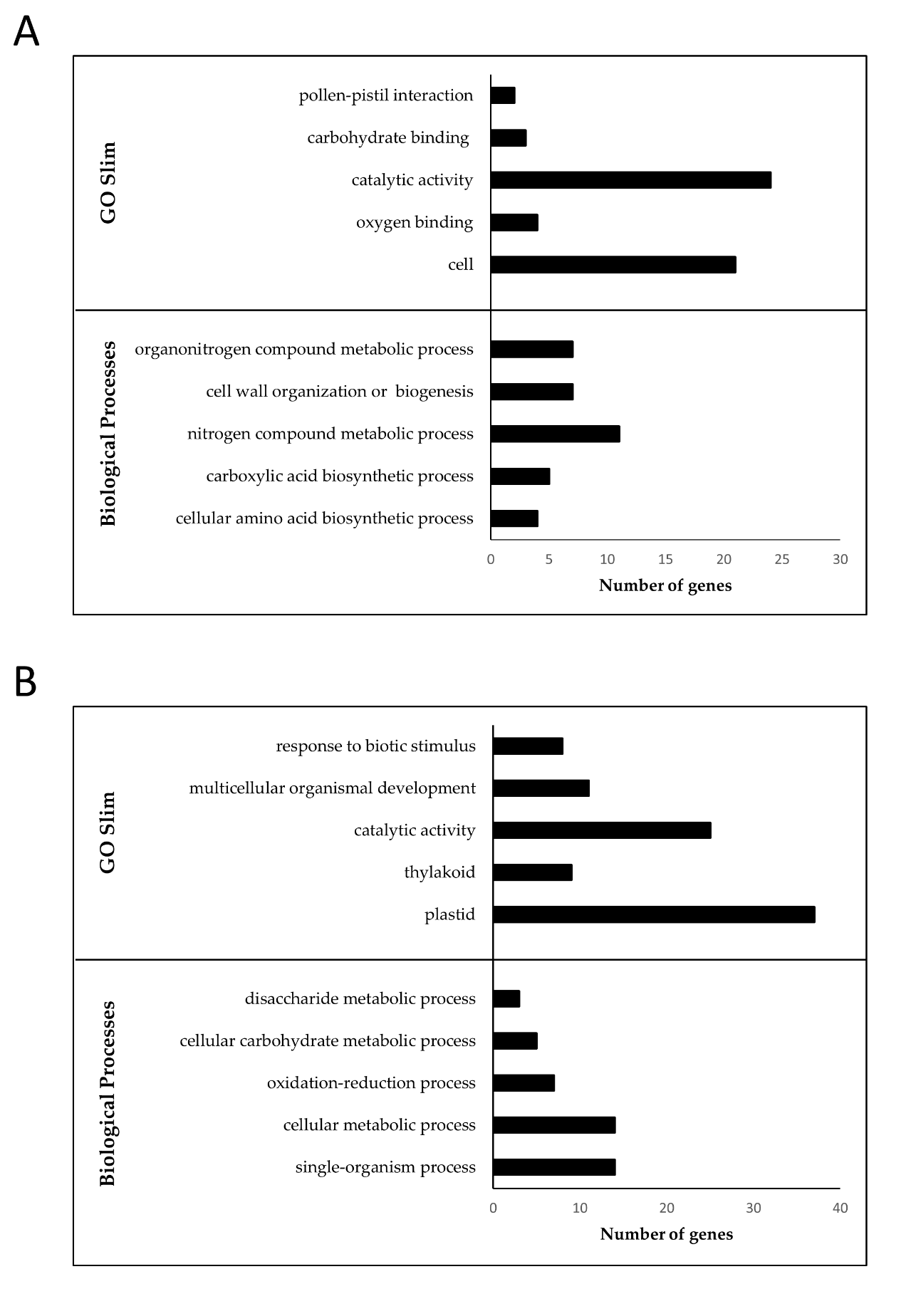
**

**Figure S12**. Major biological processes and over-represented GO Slim descriptions of drought responsive FL-M14 (A) and FL-M16 (B) in flag-leaf tissue under RDS.

**
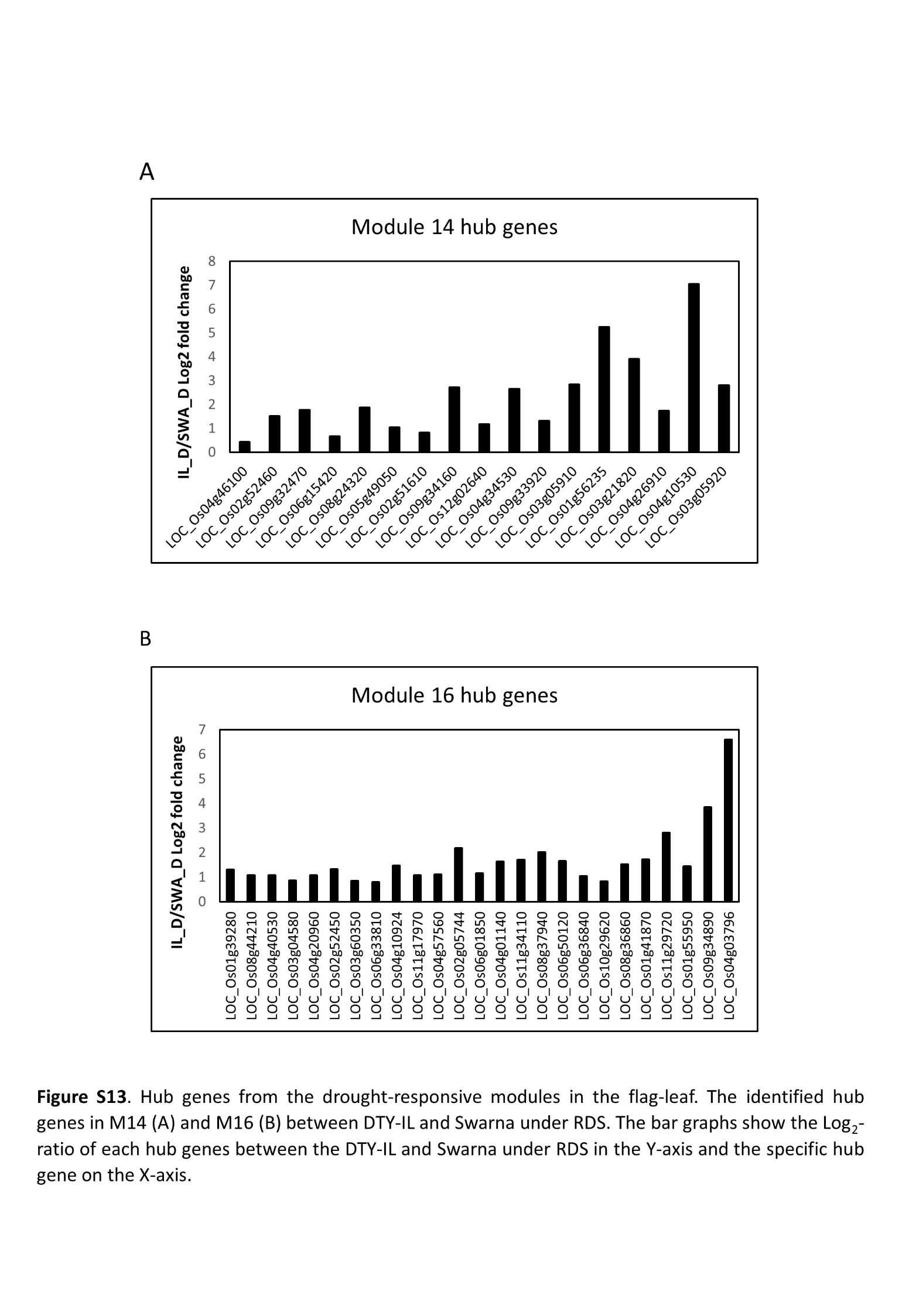
**

**Figure S13**. Hub genes from the drought-responsive modules in the flag-leaf. The identified hub genes in FL-M14 (A) and FL-M16 (B) between DTY-IL and Swarna under RDS. The bar graphs show the Log_2_-ratio of each hub genes between the DTY-IL and Swarna under RDS in the Y-axis and the specific hub gene on the X-axis.


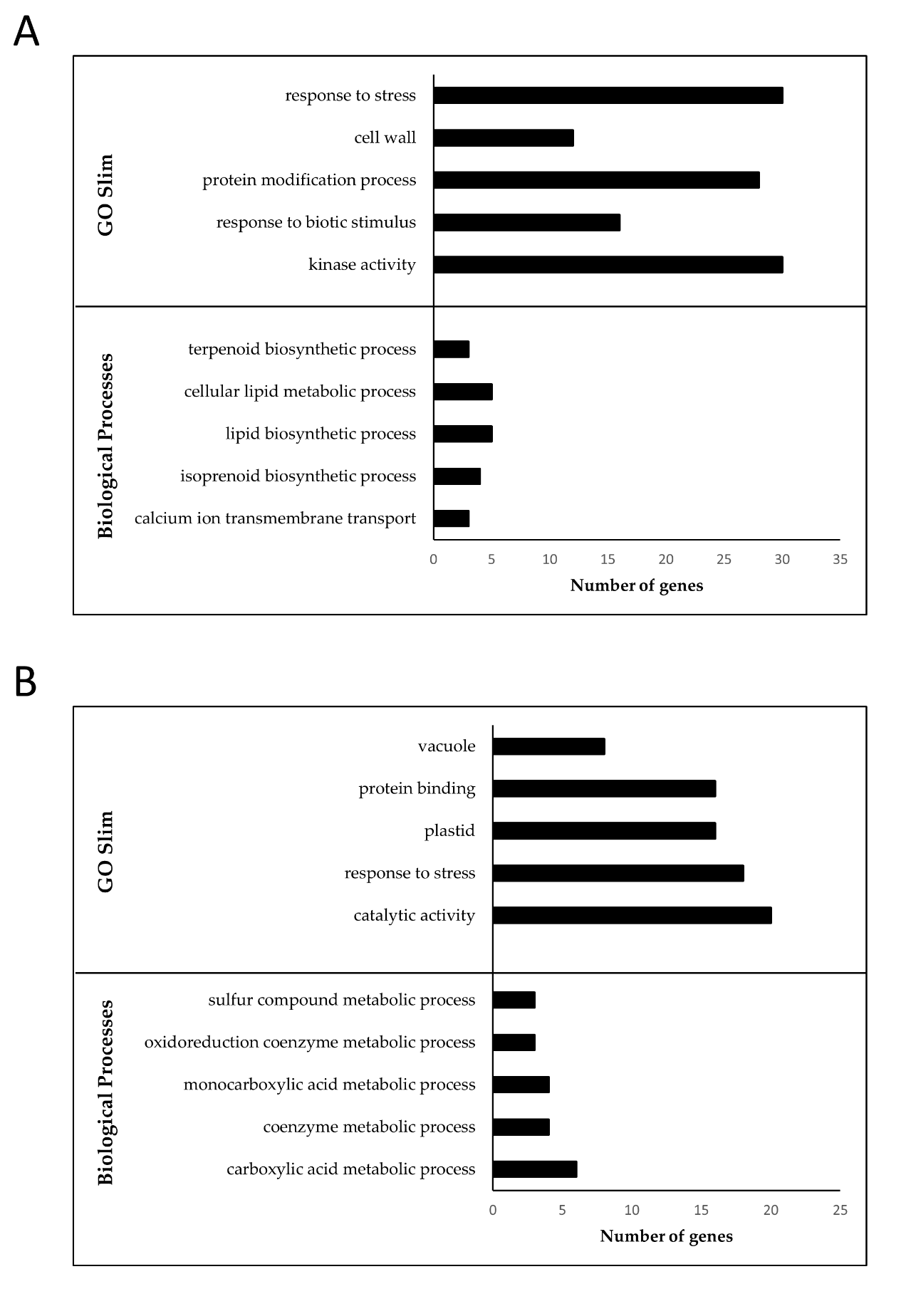


**Figure S14**. Major biological processes and over-represented GO Slim descriptions of drought responsive P-M10 (A) and P-M15 (B) in panicle tissue under RDS.


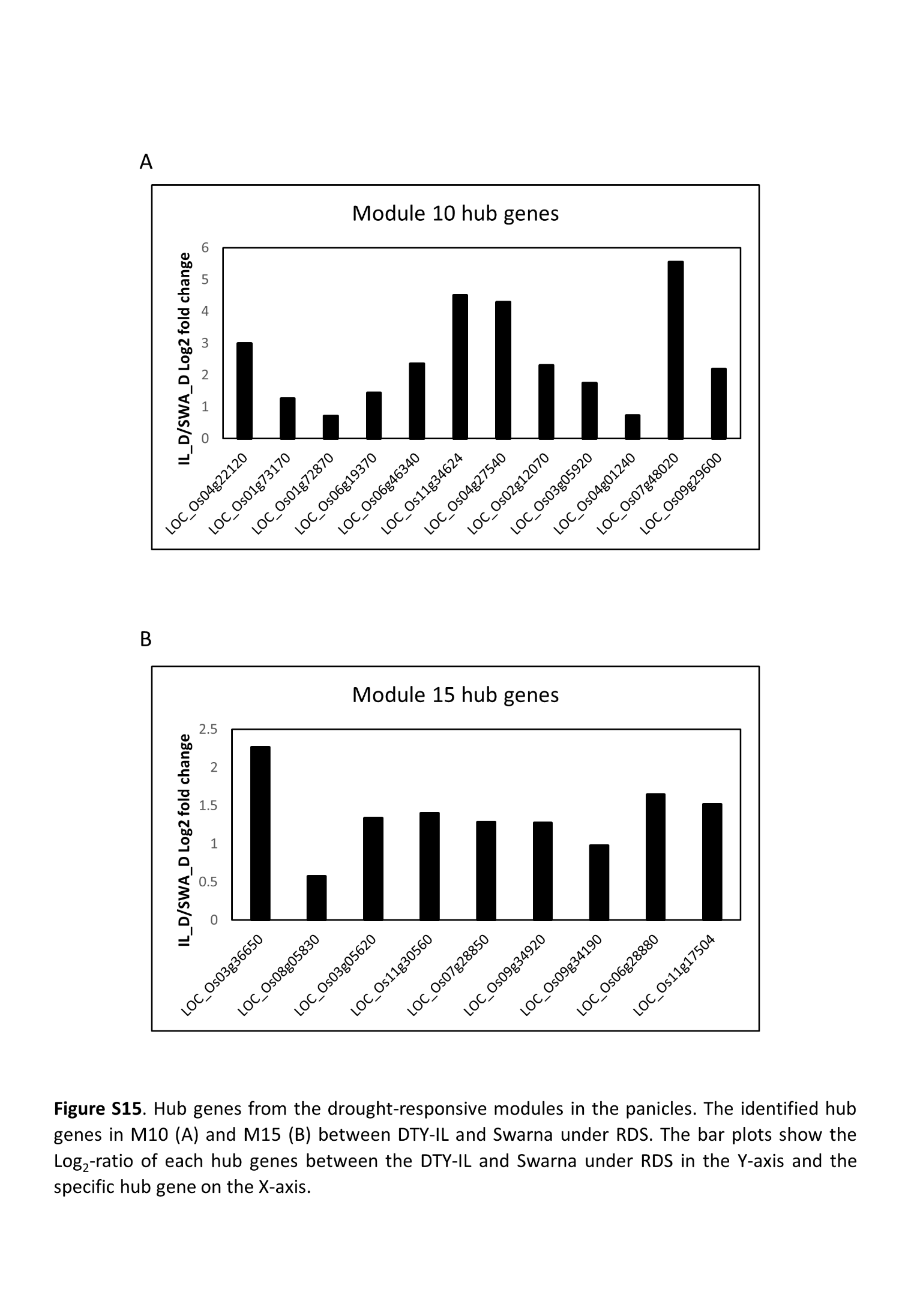


**Figure S15**. Hub genes from the drought-responsive modules in the panicles. The identified hub genes in P-M10 (A) and P-M15 (B) between DTY-IL and Swarna under RDS. The bar plots show the Log_2_-ratio of each hub genes between the DTY-IL and Swarna under RDS in the Y-axis and the specific hub gene on the X-axis.


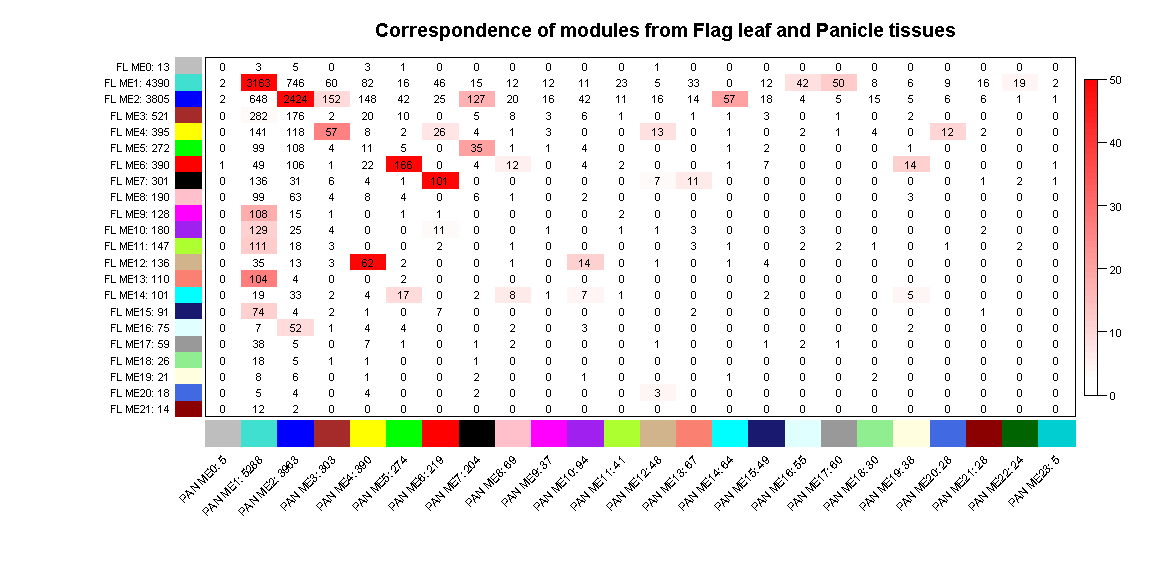
**Figure S16**. The tissue-specific expression under RDS. Consensus network matrix for the flag-leaf and panicle networks. The X-axis represents all the 23 panicle modules with the corresponding number of genes for each module. The Y-axis represents all the 21 flag-leaf modules with the corresponding number of genes for each module. Numbers beside the module number are the total gene counts for that module. Numbers inside the matrix are the number of genes common between the flag-leaf and panicle modules. Red numbers have significant overlap in gene count based on Fisher’s exact test with the –log(p) of the p-value encoding the coloring (0 = -log(1) and 50 = -log(1E-50)). FL ME = Flag-leaf module; PAN ME = Panicle module. ME0 = grey module for both tissues that was not included as the genes in this module don’t fit anywhere else.

**
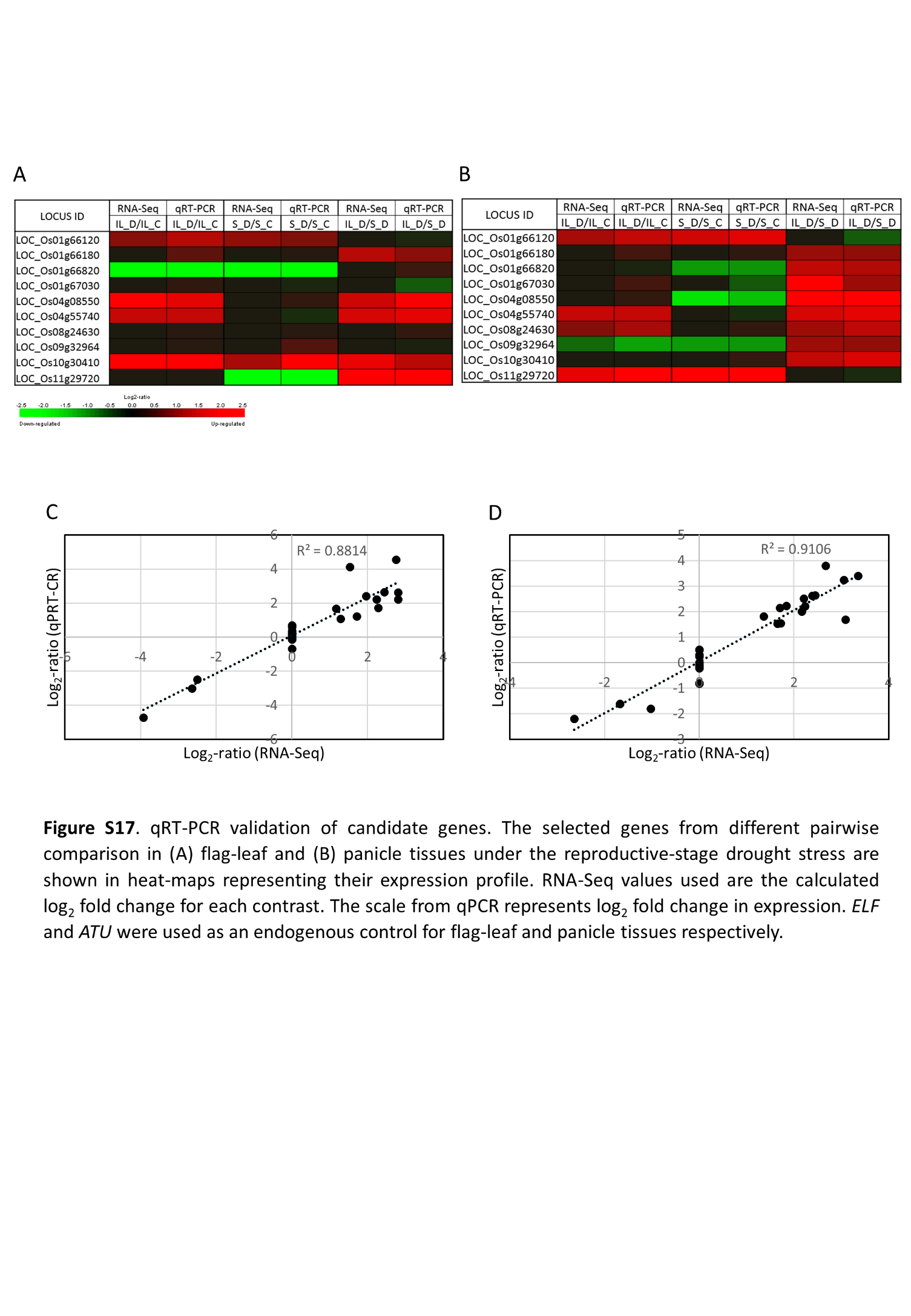
**

**Figure S17**. qRT-PCR validation of candidate genes. The selected genes from different pairwise comparison in (A) flag-leaf and (B) panicle tissues under the reproductive-stage drought stress are shown in heat-maps representing their expression profile. RNA-Seq values used are the calculated log_2_ fold change for each contrast. The scale from qPCR represents log_2_ fold change in expression. *ELF* and *ATU* were used as an endogenous control for flag-leaf and panicle tissues respectively.


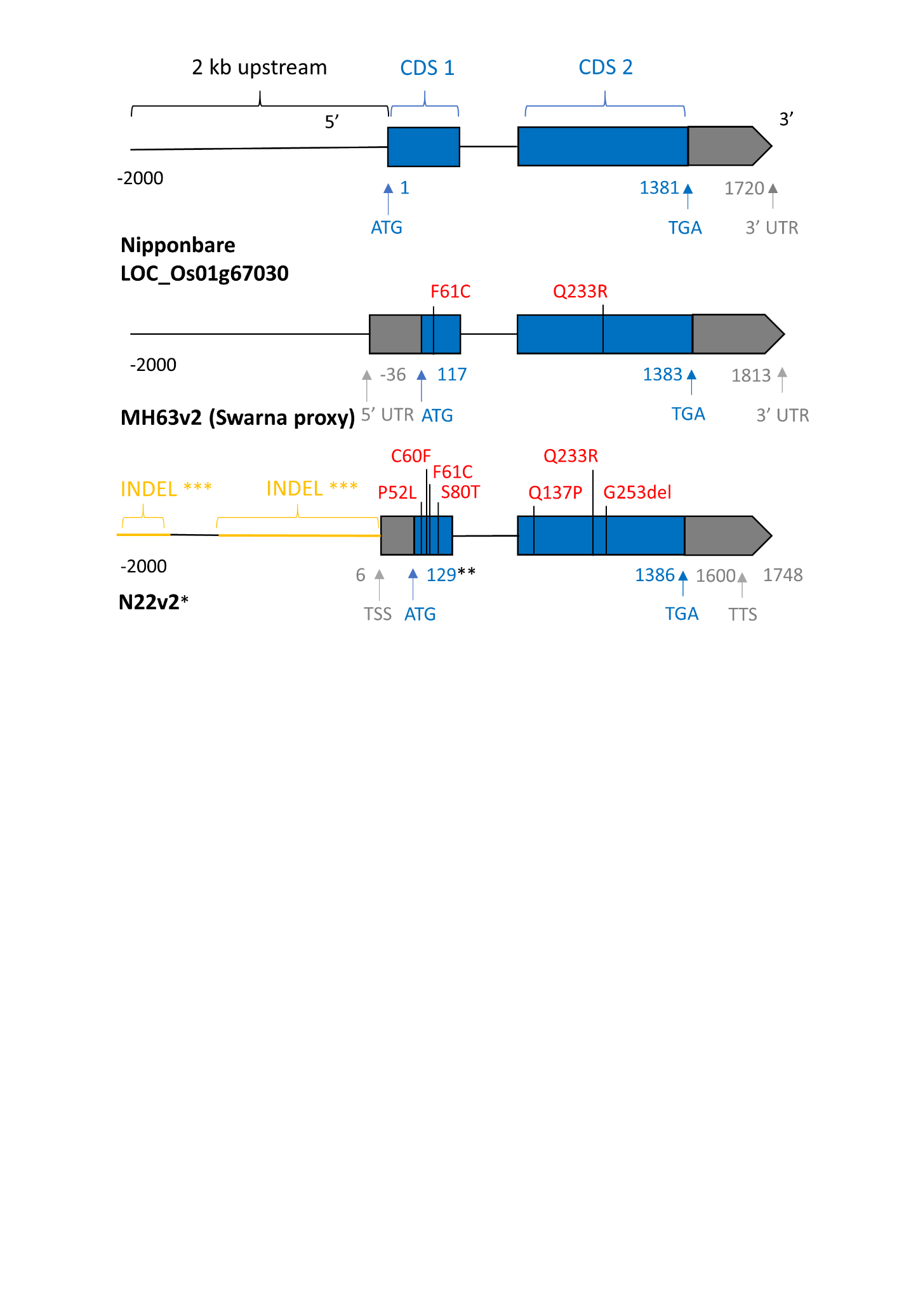


**Figure S18.** Comparison of gene structure including the 2-kb upstream region of AuxRe. The 2-kb upstream and full-length sequence of AuxRe in Nipponbare was used to lift over the corresponding sequences of AuxRe in MH63v2 and N22v2 genomes via blastn. Organization of exons (blue box), introns, and UTRs (gray box) of AuxRe (LOC_Os01g67030) gene in the gene body and the 2-kb upstream sequences of the gene are shown for Nipponbare, MH63v2 (an indica reference genome used as a proxy for the closely related Swarna), and N22v2, representing *Japonica, Indica,* and *Ausboro* genomes respectively. The alignment of the promoter and the full-length genomic region was done using Blastn. Gene structure annotation was present in Nipponbare (RGAP 7) and MH63v2 (RIGW) while it was predicted in the N22v2 using FGENESH. Non-synonymous SNP’s (in color red texts) were determined relative to the Nipponbare sequence. *FGENESH prediction; **Different start codon in FGENESH prediction; ***Alignment result through Blastn in NCBI Blast. CDS = coding sequence; UTR = untranslated region; ATG = start codon; TGA = stop codon; TSS = transcription start site; TTS = transcription termination site.


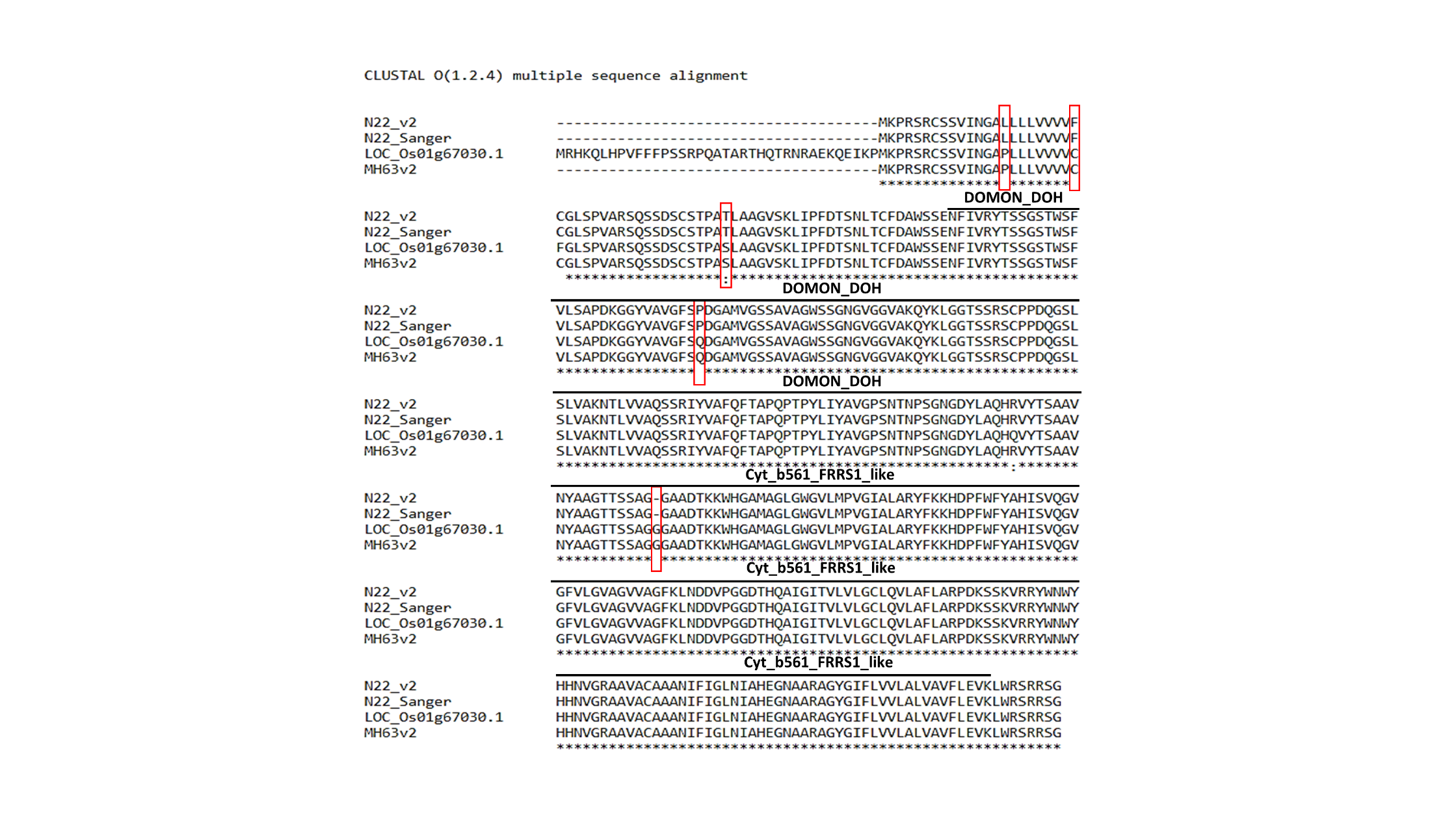


**Figure S19**. Multiple peptide sequence alignment of LOC_Os01g67030 in Nipponbare, MH63v2, N22_v2, and a clone amplified from N22 (N22_Sanger). Nipponbare sequence was obtained from the Rice Genome Annotation Project (RGAP 7), while MH63v2 sequence was obtained from the Rice Information Gateway (RIGW). Predictions in N22v2 were made in FGENESH. Protein alignment was done using Clustal Omega using default parameters with ClustalW output format. Conserved domains search was done through CD-search in NCBI with default settings. The DOMON_DOH and Cyt_b561_FRRS1_like conserved domains were underlined. Note: The Sanger sequencing result of N22 validates the SNPs and Indels in the predicted AA of N22_v2. Nonsynonymous SNPs and a Glycine deletion unique to N22v2 were highlighted (red rectangular box). *, identical residues.


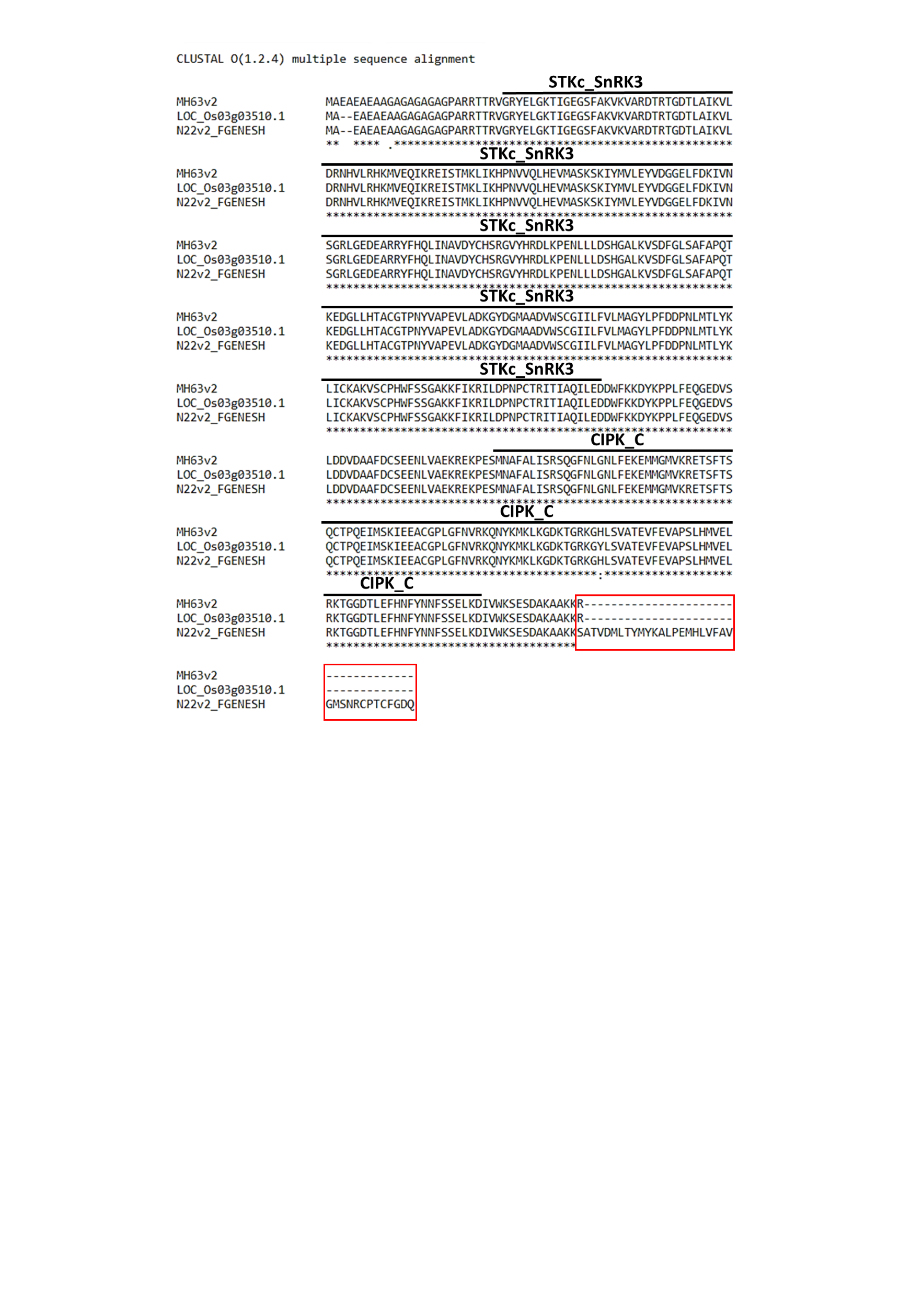


**Figure S20**. Multiple peptide sequence alignment of LOC_Os03g03510 in Nipponbare, MH63, and N22 sequences. Nipponbare sequence was obtained from the Rice Genome Annotation Project (RGAP 7), while MH63v2 sequence was obtained from the Rice Information Gateway (RIGW). Predictions for N22v2 were made in FGENESH. Protein alignment was done using Clustal Omega using default parameters with ClustalW output format. Conserved domains search was done through CD-search in NCBI with default settings. The STKc_SnRK3 and CIPK_C conserved domains were underlined. The extended C-terminal region (red rectangular box) in N22v2 was highlighted. *, identical residues.

Redox HS

Sec Met

Protein TO

Detox

**Fertile**

Source-

Sink-

Signalling

**Sterile**

Starvation

Source-

Sink-

Signalling

A

B

**Figure S21**. Model of suggested DTY-IL dependent drought tolerance mechanism.

1. In DTY-IL in flag leaves (green), maintained and drought-adapted cell wall homeostasis (solid green bricks) under drought (no water and sun icons) supports sustained photosynthetic activity (large green chloroplast icon). This, in turn, allows for strong source strength and the allocation of energy and carbon in the form of photo-assimilates to emerging panicles. A strong sink is established, and energy and carbon available for Redox-homeostasis  (Redox HS), protective secondary metabolism (Sec Met), high rates of protein turnover (Protein TO) and detoxification (Detox) to enable the emerging panicle to cope with drought stress and remain fertile, ultimately leading to successful grain filling and drought-tolerant yield (DTY). This is proposed to be in part accomplished through efficient source-sink signaling (blue arrow) suggested to be modulated through the identified candidate genes.
2. In Swarna flag leaves (green), failure to maintain cell wall integrity (wavy green bricks) under drought results in leaf rolling and impaired photosynthetic activity (small chloroplast icon). This, in turn negatively affects source strength and severely limits the allocation of energy and carbon in the form of photo-assimilates to emerging panicles. Consequently, the merging panicle is in starvation mode and fails to establish a strong sink. There is not sufficient carbon energy available to successfully counter drought stress, and the results are sterility and failure to fill grain and yield under drought. This is proposed to be in part due to failure to modulate source-sink signaling in response to drought stress (yellow lightning across blue arrow) and unfavorable alleles on the identified candidate genes suggested to contribute.
